# Evaluation of the significance of polyamines and their oxidases in the aetiology of human cervical carcinoma.

**DOI:** 10.1038/bjc.1995.485

**Published:** 1995-11

**Authors:** C. Fernandez, R. M. Sharrard, M. Talbot, B. D. Reed, N. Monks

**Affiliations:** Institute for Cancer Studies, University of Sheffield Medical School, UK.

## Abstract

The risk of cancer of the cervix is linked with sexual behaviour. Although infectious agents such as human papillomaviruses (HPVs) are implicated, these alone may be insufficient to induce the disease. We have investigated the potential role of oxidation products of the polyamines spermine and spermidine and the diamine putrescine in seminal plasma (SP) as co-factors in the development of cervical cancer. These amines are oxidised by polyamine oxidase (PAO) and diamine oxidase (DAO) to generate oxygen radicals and hydrogen peroxide, reactive aldehydes and acrolein, which are likely to exert local mutagenic, cytotoxic and immunosuppressive effects in vivo. Using a chemiluminescence assay, we determined the levels of these amines in 187 samples of SP. Spermine plus spermidine, as substrates for PAO, were present in a range equivalent to 0-4.8 mg ml-1 spermine. Putrescine, as a substrate for DAO, was detectable in only 4 of 40 samples assayed (range 0-168 micrograms ml-1) and constitutes a minor component of the oxidisable content of SP. Cervical mucus (126 samples) was assayed for the presence of PAO and DAO. Both enzymes were present in 14.3% of the samples, PAO only in 21.4%, DAO only in 15.1% and neither enzyme in 49.2%. PAO levels ranged from 0 to 0.828 pmol peroxide generated min-1 mg-1 mucus and DAO levels ranged from 0 to 7.0 pmol peroxide generated min-1 mg-1 mucus. These results suggest that sexual activity in the absence of physical barrier contraception may lead to the generation of mutagenic and immunosuppressive polyamine oxidation products within the female genital tract. We thus propose that women with high levels of PAO and/or DAO in their cervical mucus may be at increased risk of cervical cancer, especially if the male partner's SP shows high polyamine levels. HPV infection may synergise with the effects of polyamine oxidation by suppressing apoptosis in keratinocytes carrying potentially oncogenic mutations, leading to the survival and proliferation of transformed cells in the cervix.


British Journal of Cancer (1995) 72, 1194-1199

© 1995 Stockton Press All rights reserved 0007-0920/95 $12.00

### Evaluation of the significance of polyamines and their oxidases in the aetiology of human cervical carcinoma

C Fernandez[1], RM Sharrard[1], M Talbot[2], BD Reed[3] and N Monks[4]

[1]*Institute for Cancer Studies, University of Sheffield Medical School, Beech Hill Road, Sheffield S10 2RX, UK;* [2]*Department of Genito-Urinary Medicine, Royal Hallamshire Hospital, Glossop Road, Sheffield S10 2JF, UK;* [3]*Department of Family Practice, University of Michigan, Ann Arbor, Michigan, USA;* [4]*Sheffield Fertility Centre, Glen Road, Sheffield S7 1RA, UK.*

**Summary** The risk of cancer of the cervix is linked with sexual behaviour. Although infectious agents such as human papillomaviruses (HPVs) are implicated, these alone may be insufficient to induce the disease. We have investigated the potential role of oxidation products of the polyamines spermine and spermidine and the diamine putrescine in seminal plasma (SP) as co-factors in the development of cervical cancer. These amines are oxidised by polyamine oxidase (PAO) and diamine oxidase (DAO) to generate oxygen radicals and hydrogen peroxide, reactive aldehydes and acrolein, which are likely to exert local mutagenic, cytotoxic and immunosuppressive effects *in vivo*. Using a chemiluminescence assay, we determined the levels of these amines in 187 samples of SP. Spermine plus spermidine, as substrates for PAO, were present in a range equivalent to 0-4.8 mg $ml^{-1}$ spermine. Putrescine, as a substrate for DAO, was detectable in only 4 of 40 samples assayed (range 0-168 $\mu g$ $ml^{-1}$) and constitutes a minor component of the oxidisable content of SP. Cervical mucus (126 samples) was assayed for the presence of PAO and DAO. Both enzymes were present in 14.3% of the samples, PAO only in 21.4%, DAO only in 15.1% and neither enzyme in 49.2%. PAO levels ranged from 0 to 0.828 pmol peroxide generated $min^{-1}$ $mg^{-1}$ mucus and DAO levels ranged from 0 to 7.0 pmol peroxide generated $min^{-1}$ $mg^{-1}$ mucus. These results suggest that sexual activity in the absence of physical barrier contraception may lead to the generation of mutagenic and immunosuppressive polyamine oxidation products within the female genital tract. We thus propose that women with high levels of PAO and/or DAO in their cervical mucus may be at increased risk of cervical cancer, especially if the male partner's SP shows high polyamine levels. HPV infection may synergise with the effects of polyamine oxidation by suppressing apoptosis in keratinocytes carrying potentially oncogenic mutations, leading to the survival and proliferation of transformed cells in the cervix.

**Keywords:** polyamines; polyamine oxidase; diamine oxidase; cervical cancer

The aetiology of cervical cancer is still the subject of debate. Strong epidemiological evidence has linked cervical cancer risk with sexual behaviour (Franco, 1991; Joffe *et al.*, 1992), suggesting the involvement of a transmissible agent. However, simple models based upon infectious agents alone, such as human papillomavirus (HPV), have been shown to be inadequate. HPV infection is still much more common than the occurrence of cervical intraepithelial neoplasia (CIN) and invasive cervical cancer (Schiffman, 1992); most women who are HPV positive show no evidence of cervical abnormalities (De Villiers *et al.*, 1987; Meanwell *et al.*, 1987; Reeves *et al.*, 1987) and only a small proportion of women with HPV develop cervical cancer (Mitchell *et al.*, 1986). Furthermore, the long latent periods (20-50 years) between HPV infection and the development of cancer (zur Hausen, 1986) suggests that HPV alone is insufficient to induce cervical cancer. A number of additional aetiological factors have been proposed as increasing the risk of developing the disease. These include heavy smoking (Winkelstein, 1990), the long-term use of oral contraceptives (Jones *et al.*, 1990; Gram *et al.*, 1992), dietary factors (Potischman, 1993), concomitant infection with herpes simplex type 2 (Hildesheim *et al.*, 1991) or other sexually transmitted diseases (Guijon *et al.*, 1985; La Vecchia *et al.*, 1986; Herrero *et al.*, 1990), and the immunosuppressed states associated with organ transplantation, pregnancy, or acquired immunodeficiency disease (Schneider *et al.*, 1987; Alloub *et al.*, 1989; Henry *et al.*, 1989).

Risk of acquisition of sexually transmitted disease correlates with sexual activity: intercourse with a large number of sexual partners increases the chance of exposure to such transmissible agents. However, risk of cervical disease is more closely related to the number of steady partners with relationships lasting more than 3 months than to the number of non-steady partners, an effect more apparent for those who had multiple steady partners at younger ages (Herrero *et al.*, 1990). This suggests the need for more prolonged or repeated exposure to a partner who carries a transmissible agent. Attention has thus recently turned to investigation of chemical components of semen which may act together with infectious agents as co-factors in the development of cervical cancer or as inducers of cancer in their own right.

Seminal plasma (SP) has potent immunosuppressive activity. A number of SP components have been demonstrated to be capable of modulating a variety of immunological functions (Quayle *et al.*, 1989; Ablin *et al.*, 1990; Kelly *et al.*, 1991; Skibinski *et al.*, 1992). The polyamines spermine, spermidine and putrescine occur at high concentrations in SP (Williams-Ashman and Lockwood, 1970); their physiological function here is unknown, although it has been suggested that they inhibit the coagulation of semen in the male urethra (see Oefner *et al.*, 1992). Polyamines have been shown to mediate immunosuppressive effects *in vitro* through their oxidation products (Allen and Roberts, 1987; Vallely *et al.*, 1988; Quan *et al.*, 1990); hydrogen peroxide, acrolein and reactive aldehydes generated by enzymic oxidation of polyamines show cytotoxic properties (Averill-Bates *et al.*, 1993) as well as the ability to cause DNA strand breaks (Henle *et al.*, 1986) and chromatid aberrations (Bouzyk and Rosiek, 1988) and to induce programmed cell death (Parchment and Pierce, 1989; Gramzinski *et al.*, 1990). The presence in the female genital tract of enzymes catalysing the oxidation of polyamines from a male partner's semen could thus lead both to local immunosuppression and to exposure of the cervical epithelial cells to genotoxic agents. Either or both of these effects could be significant in the initiation and progression of cervical cancer.

Correspondence: RM Sharrard
Received 9 January 1995; revised 12 June 1995; accepted 20 June 1995

The relevance of polyamine oxidation *in vivo* to the aetiology of cervical malignancy has not previously been examined. The present study was undertaken to investigate the possible involvement of polyamines present in SP and of polyamine oxidation enzymes in cervical mucus as co-factors in the development of cervical carcinoma.

### Materials and methods

A total of 120 seminal plasma samples were donated from males attending the Sheffield Fertility Centre, Sheffield; an additional 67 samples were obtained from the partners of women in a case-control study of vaginitis at the Department of Family Practice, University of Michigan Medical School, Ann Arbor, Michigan, USA. Fresh ejaculates were centrifuged and the supernatants stored at −80°C until assayed. No decrease in oxidisable polyamine level was observed in samples stored in this way for several months.

One hundred and twenty-six cervical mucus samples were obtained from women attending the Department of Genito-Urinary Medicine, Royal Hallamshire Hospital, Sheffield. The age range was 16–56 years (median 23 years). The specimens were collected during routine examination of the cervix using a disposable plastic loop which was used to transfer mucus to preweighed vials containing 0.5 ml of 10 mM Tris-HCl pH 7.5/150 mM sodium chloride. The vials were reweighed to estimate the weight of mucus collected (10–50 mg). The samples were stored overnight at 4°C and assayed the following day.

Spermine tetrahydrochloride, diamine oxidase, collagenase, hyaluronidase, microperoxidase, luminol and hydrogen peroxide were purchased from Sigma, Poole, Dorset.

Polyamine oxidase (PAO) was purified from newborn calf serum on a 30 × 1 cm column of DEAE-Sepharose Fast Flow (Pharmacia) using a Pharmacia FPLC apparatus. The column was eluted with a gradient of 0–0.1 M sodium chloride in 75 ml, then 0.1–0.5 M sodium chloride in 125 ml, then 50 ml of 0.5 M sodium chloride. Fractions containing PAO were identified using a modified form of the colorimetric assay described by Aarsen and Kemp (1964), using spermidine as substrate. The PAO-containing fractions were pooled for use in the assay of spermine and spermidine.

### *Detection of polyamines in seminal plasma*

Polyamine (spermine and spermidine) levels were determined using the chemiluminescence assay described by Fernandez *et al.* (1994) as follows: SP samples were diluted 1:60 in reaction buffer (1 mM glycine hydrogen chloride pH 7.4/0.01 mM sodium carbonate). An aliquot of 20 μl of diluted SP was mixed with 1 ml of reaction buffer in a stoppered cuvette, 10 μl of purified PAO was added, and the cuvette was incubated at room temperature for 1 h. The reaction was completed by the addition of 20 μl of 0.9 mg ml$^{-1}$ luminol (final concentration 18 μg ml$^{-1}$) and 0.1 ml of 0.1 mg ml$^{-1}$ microperoxidase (final concentration 10 μg ml$^{-1}$). Light emitted was measured using a BioOrbit 1251 luminometer with BioOrbit 1257 software ('Phagocytosis' programme) or a BioOrbit 1250 luminometer linked to a standard chart recorder. Integral values were calibrated against those obtained using known spermine standards. Putrescine was assayed by substituting DAO for PAO to a final concentration of 15 μg ml$^{-1}$ and incubating at 37°C for 24 h.

### *Detection of polyamine and diamine oxidases in cervical mucus*

Preweighed cervical mucus samples were incubated with 10 U ml$^{-1}$ of collagenase and 200 μg ml$^{-1}$ hyaluronidase for 2–3 h at room temperature. Separate experiments were carried out to determine that this pretreatment did not affect the levels of polyamine or diamine oxidase present in the samples (results not shown). The samples were then diluted 1:3 in 10 mM Tris-HCl pH 7.4/150 mM sodium chloride and assayed for the presence of PAO and DAO using a modification of the chemiluminescence assay as follows: 15 μl of diluted cervical mucus was incubated in the presence of 6 μg ml$^{-1}$ spermine for 24 h at 37°C in a stoppered cuvette for the detection of PAO or 30 μg ml$^{-1}$ putrescine for 1 h at 37°C for the detection of DAO. Microperoxidase (final concentration 10 μg ml$^{-1}$) and luminol (final concentration 18 μg ml$^{-1}$) were added and the samples read as above. Integral values were calibrated against standards containing known concentrations of hydrogen peroxide. Enzyme activities were expressed as pmols hydrogen peroxide generated min$^{-1}$ mg$^{-1}$ cervical mucus.

### *Estimation of the generation of hydrogen peroxide by interaction of seminal plasma with cervical mucus*

Eleven samples of cervical mucus were incubated with 10 U ml$^{-1}$ of collagenase and 200 μg ml$^{-1}$ hyaluronidase for 2–3 h at room temperature as above. The samples were diluted 1:3 in 10 mM Tris-HCl pH 7.4/150 mM sodium chloride and 15 μl aliquots were assyed for PAO activity using either 6 μg ml$^{-1}$ spermine tetrahydrochloride or seminal plasma diluted to give a final oxidisable polyamine concentration of 6 μg ml$^{-1}$. The reactions were incubated for 24 h at 37°C in a stoppered cuvette and assayed for hydrogen peroxide as described above.

### Results

### *Polyamine levels in seminal plasma samples*

The total PAO-oxidisable polyamine content of 187 seminal plasma samples was determined (Figure 1a) and found to fall within a range equivalent to 0.0–4.8 mg ml$^{-1}$ (0–13.8 mM) spermine. The median value was 0.6 mg ml$^{-1}$ (1.7 mM), the upper quartile 1.2 mg ml$^{-1}$ (3.5 mM) and the lower quartile 0.2 mg ml$^{-1}$ (0.6 mM). These values are similar to those determined using alternative methods designed for detecting specific polyamines (see Jänne *et al.*, 1973; Jakobsen *et al.*, 1989; Oefner *et al.*, 1992). No correlation was found between the density and percentage motility of sperm in the original semen samples and the polyamine content of the plasma as determined by the assay. We have previously reported evidence that individual males maintain relatively constant

**Figure 1** PAO-oxidisable polyamine levels in (a) seminal plasma samples from 187 males in Sheffield and the USA, (b) 62 males whose female partners have a normal cervix and (c) 12 males whose female partners have abnormalities of the cervix (dysplasia and various stages of cervical intraepithelial neoplasia pooled together), as determined by the chemiluminescence assay. Each dot represents an individual seminal plasma sample, the box represents the upper and lower quartile values and the line within the box represents the median value.

levels of PAO-oxidisable polyamines in SP over time (Fernandez *et al*., 1994).

Putrescine levels were determined separately, using DAO, in 40 seminal plasma samples. Only four of these samples contained putrescine at concentrations detectable by this assay (12, 24, 42 and 168 $\mu g\ ml^{-1}$); this diamine thus constitutes a minor component of the total oxidisable polyamine content of seminal plasma.

### *Relationship between polyamine levels and abnormalities of the cervix*

Details of the state of the cervix from women attending the Sheffield Fertility Centre whose partners provided a semen sample were available for some, but not all, of the analyses. Figure 1b shows the distribution of PAO-oxidisable polyamines in the SP of 62 males whose female partners showed no cervical abnormalities (range 0–3.1 $mg\ ml^{-1}$; median 0.7, upper quartile 1.2, lower quartile 0.3 $mg\ ml^{-1}$) and Figure 1c shows the values of PAO-oxidisable polyamines in the SP of 12 males whose female partners had dysplastic changes or any stage of CIN (range 0.1–3.0 $mg\ ml^{-1}$; median 0.9, upper quartile 1.2, lower quartile 0.2 $mg\ ml^{-1}$). Statistical analysis by the Mann–Whitney *U*-test did not demonstrate any significant correlation between polyamine content of SP samples from males and the state of the cervix in their respective female partners. However, since the number of cases showing abnormalities of the cervix was small ($n = 12$), no definitive conclusion could be drawn on the relevance of polyamine concentration to the occurrence of abnormalities of the cervix.

Extensive clinical information was collected from both male and female partners ($n = 67$) attending the Department of Family Practice, University of Michigan Medical School, MI, USA. No correlation could be identified between the PAO-oxidisable polyamine content of SP samples from males as determined by the chemiluminescence assay and the occurrence of genital warts, HPV infection, other genital infections including Herpes, *Trichomonas*, *Chlamydia* and *Candida*, cervical cancer or abnormalities of the cervix of respective partners. There was also no correlation between polyamine content and any other parameter concerning the male, including smoking, ethnic group, previous history of genital warts and genital infections such as Herpes, *Trichomonas*, *Chlamydia* and *Candida*, vasectomy, or circumcision, as determined by statistical analysis of variance (ANOVA).

### *Diamine oxidase and polyamine oxidase levels in cervical mucus samples*

One hundred and twenty-six cervical mucus samples were assayed for the presence of DAO and PAO (Figure 2a and b). DAO alone was detected in 19/126 (15.1%) of the samples, PAO alone in 27/126 (21.4%), DAO plus PAO in

**Figure 2** (**a**) DAO levels detected in 126 cervical mucus samples. The figure represents the level of DAO in each sample as pmol hydrogen peroxide generated $min^{-1}\ mg^{-1}$ cervical mucus as determined using the chemiluminescence assay. (**b**) PAO levels detected in 126 cervical mucus samples. The figure represents the level of PAO in each sample as pmol hydrogen peroxide generated $min^{-1}\ mg^{-1}$ cervical mucus as determined using the chemiluminescence assay.

18/126 (14.3%) and neither enzyme in 62/126 (49.2%). Levels of DAO varied from undetectable to 7.0 pmol peroxide generated $min^{-1}$ $mg^{-1}$ mucus (Figure 2a); the median of the values in which DAO activity was detectable was 0.36 pmol $min^{-1}$ $mg^{-1}$ (upper quartile 0.83, lower quartile 0.23). PAO levels ranged from undetectable to 0.828 pmol peroxide generated $min^{-1}$ $mg^{-1}$ mucus (Figure 2b); the median of the values in which PAO was detectable was 0.038 pmol peroxide generated $min^{-1}$ $mg^{-1}$ mucus (upper quartile 0.079, lower quartile 0.012). Statistical analysis of the DAO and PAO levels in individual women who expressed detectable levels of both enzymes showed evidence of a linear relationship between DAO and PAO values (linear correlation coefficient 0.962; Figure 3).

### *Generation of hydrogen peroxide in the interaction between seminal plasma and cervical mucus*

In order to test whether cervical mucus polyamine-oxidising enzymes are still active in the presence of seminal plasma, estimations of the levels of hydrogen peroxide generated by cervical mucus oxidases were carried out using seminal plasma as a source of substrate in place of purified spermine.

**Figure 3** Distribution of 126 cervical mucus samples containing PAO and/or DAO, as determined by the chemiluminescence assay. DAO and PAO levels are represented as pmol hydrogen peroxide generated $min^{-1}$ $mg^{-1}$ cervical mucus and are plotted on a log scale for clarity. Cervical mucus samples without the detectable presence of either PAO or DAO ($n = 62$) are represented by a single dot at the intersection in the above graph, 19 mucus samples contain DAO only, 27 mucus samples contain PAO only and 18 mucus samples contain both enzymes.

**Table I** Eleven cervical mucus samples assayed for the presence or absence of PAO

| *Cervical mucus sample* | *PAO (pmol hydrogen peroxide generated $min^{-1}$ $mg^{-1}$ mucus)* | |
|---|---|---|
| | *Spermine tetrachloride* | *Seminal plasma* |
| 1 | 0.105 | 0.896 |
| 2 | 0.054 | 1.491 |
| 3 | 0.004 | 0 |
| 4 | 0.002 | 0.538 |
| 5 | 0 | 0 |
| 6 | 0.044 | 1.101 |
| 7 | 0.116 | 0.666 |
| 8 | 0 | 0 |
| 9 | 0.089 | 0.683 |
| 10 | 0.021 | 0.062 |
| 11 | 0.037 | 0.688 |

Either spermine tetrachloride (6 $\mu g$ $ml^{-1}$ final concentration) or seminal plasma (6 $\mu g$ $ml^{-1}$ PAO-oxidisable polyamines) are used as substrate in the chemiluminescence assay. Values represent the level of PAO as pmol hydrogen peroxide generated $min^{-1}$ $mg^{-1}$ cervical mucus.

The results are shown in Table I. With the exception of one specimen (sample 3), which showed a low level of PAO activity with spermine as substrate but no activity with SP, all the cervical mucus samples which showed detectable PAO activity with spermine as substrate generated hydrogen peroxide when incubated with seminal plasma, indicating that semen does not contain significant levels of inhibitors of polyamine oxidases. Interestingly, the levels of hydrogen peroxide generated by cervical mucus in reaction with seminal plasma were higher than those generated with spermine alone, indicating either that the polyamines in seminal plasma are better substrates for the enzyme (perhaps owing to the presence of an enzyme co-factor) or that the hydrogen peroxide generated is stabilised by a component of semen.

### Discussion

Oxidisable polyamine levels were determined in 187 samples of seminal plasma using a novel chemiluminescence assay (Fernandez *et al.*, 1994). The results were similar to those determined by other published methods: PAO-oxidisable polyamine levels ranged from undetectable to as high as 4.8 mg $ml^{-1}$. Only 4 out of 40 samples displayed measurable levels of putrescine, with the highest concentration being 168 $\mu g$ $ml^{-1}$. Putrescine has previously been found in trace amounts in SP samples, with levels up to 200 $\mu g$ $ml^{-1}$ (Jakobsen *et al.*, 1989). In the context of risk factors for cervical carcinoma, the total content of oxidisable polyamine in SP is probably more relevant than individual polyamine concentrations.

In a previous study (Fernandez *et al.*, 1994) we showed that individual men maintain high or low levels of SP polyamines over time: oxidisable polyamine concentrations remained relatively constant in individual males when semen samples were collected following 2 days abstinence. In the present study we found no significant difference in the range of polyamine levels between SP samples from normospermic and oligospermic semen. Although aspermic semen has been shown to contain lower spermine levels than normospermic (Shohat *et al.*, 1989; Singer *et al.*, 1989), Shohat *et al.* did not find any significant differences in spermine content between oligo- and normospermic semen.

The immunosuppressive and cytotoxic effects of polyamines in SP are largely dependent on the products of their oxidation by PAO and/or DAO. We found measurable levels of one or both of these enzymes in over half of the 126 cervical mucus samples assayed. The presence of these enzymes within the female genital tract has not been reported previously, except for the detection of DAO in vaginal secretions as a marker for ruptured amnionic membranes (Bank *et al.*, 1991; Broe *et al.*, 1992). The variations in PAO and DAO expression between individuals may be genetically inherent, or may simply reflect different time points within the menstrual cycle (van der Linden *et al.*, 1992). In addition, PAO and DAO activity in the cervical mucus of some individuals might be inhibited by serum components diffusing into the fluids surrounding the cervix (Quan *et al.*, 1991). The pattern of expression of the two enzymes shown in Figure 3 suggests that the enzymes may be coordinately regulated when both are present, but that the presence or absence of each is independent of the other. Further studies are required to determine whether enzyme levels are constitutively linked and whether they remain constant within individual females through the menstrual cycle and over longer time periods.

Mixing of SP with cervical mucus *in vitro* leads to the generation of high levels of hydrogen peroxide. PAO and DAO in mucus are thus fully active against polyamines in SP. During intercourse in the absence of physical barrier contraception, when SP comes into contact with cervical mucus, polyamines in the SP will be oxidised to generate hydrogen peroxide, aldehydes and other toxic products. Complete mixing of SP with an equal volume of cervical mucus containing polyamine oxidase activity of 1 pmol $min^{-1}$ $mg^{-1}$ could lead to micromolar levels of peroxide after

2 min (in the absence of peroxidases). The effects of exposing cells to peroxide and oxygen radicals include random DNA strand breaks (Henle *et al*., 1986) and chromatid aberrations (Bouzyk and Rosiek, 1988), as well as inhibition of cellular proliferation (Averill-Bates *et al*., 1993) and suppression of immune functions (Alexander and Anderson, 1987). Programmed cell death, which is triggered by p53-dependent genomic surveillance mechanisms, is also induced by peroxide (Parchment and Pierce, 1989; Gramzinski *et al*., 1990), presumably as a result of DNA damage. Apoptosis should protect the cervix from the potentially dangerous effects of individual cells containing mutated oncogenes or tumour-suppressor genes. However, the HPV E6 and E7 genes interact with p53 and RB-1 proteins respectively (Chen and Defendi, 1992; Scheffner *et al*., 1992; Stirdivant *et al*., 1992), and thus HPV infection may interfere with induction of programmed cell death in infected cells, enhancing the survival of precancerous clones. Lowered local immune surveillance resulting from the immunosuppressive effects of polyamine oxidation products may also assist in this survival. We have shown that exposure of keratinocytes *in vitro* to hydrogen peroxide at micromolar levels induces apoptosis, while exposure to higher levels (100–200 μM) leads to cell death through direct toxicity; transfection of cells with the E6 gene of HPV18 suppressed the apoptotic component of this response, while death through direct toxicity was unaffected (RM Sharrard, C Fernandez and VB Watt, manuscript in preparation).

A synergistic interaction may thus occur in women who express (sporadically or constitutively) high levels of polyamine-oxidising enzymes, who harbour cervical HPV and who have regular unprotected sex with one or more partners whose semen contains moderate or high levels of polyamines, leading to increased risk of cervical cancer. This hypothesis does not require persisent HPV infection for the full period between the initiation of the tumorigenic process and the development of clinically detectable cancer; indeed, HPV infections have been found to persist for shorter times than once thought (Hildesheim *et al*., 1994). HPV infection would inhibit apoptosis in cervical cells at the time of the initial genomic damage, leading to their inappropriate survival. A proportion of these cells might then acquire further damage, either through further exposure to environmental mutagens or through genomic instability resulting from the initial damage (together with the effects of other HPV genes). Once a certain level of genomic damage had been established, especially if the regulation of proliferation *vs* apoptosis had been disrupted, HPV could be lost without reversion of the cells to normality.

In the present study, examination of the relationship of diverse polyamine levels in SP in relation to the presence of cervical abnormalities did not demonstrate any correlation between oxidisable polyamine levels in the male and the presence of cervical abnormalities in the female partner. However, the physiological effects of initial carcinogenic events may take 20 years to become clinically manifest, and demonstration of the mechanisms related to these events will require long-term prospective investigations. We are presently carrying out a larger study of PAO and DAO levels in women with and without cervical disease in order to determine whether high levels of one or both enzymes are at increased risk of dysplasia or neoplasia of the cervix. Our laboratory is also developing *in vitro* model systems for assessment of the potential tumorigenic synergy between seminal plasma polyamines, the presence of oxidases, and the expression within target keratinocytes of HPV E6 and E7 genes. If this synergy were proven, women who express high levels of the oxidases could be advised to use physical barrier methods of contraception to reduce their risk of cervical cancer. Screening sexually active women for the presence of PAO and DAO with the relatively simple assay system used in the present study (for example, simultaneously with routine cervical smear testing) could thus prove valuable in identifying and counselling women who may be at increased risk of cervical cancer.

**Acknowledgements**
This work was supported by the Yorkshire Cancer Research Campaign. We would like to thank all the staff of the Department of Genito-Urinary Medicine, Royal Hallamshire Hospital, Sheffield, for their unflagging help and co-operation in collecting the samples and Andrew Kyprianou and Coomaren Vencatasawmey for statistical analysis.

**References**

AARSEN PN AND KEMP A. (1964). Rapid spectrophotometric micromethod for determination of histaminase activity. *Nature*, **204**, 1195.

ABLIN RJ, BARTKUS JM AND POLGAR J. (1990). Effect of human seminal plasma on the lytic activity of natural killer cells and presumptive identification of participant macromolecules. *Am. J. Reprod. Immunol. Microbiol.*, **24**, 15–21.

ALEXANDER NJ AND ANDERSON DJ. (1987). Immunology of semen. *Fertil. Steril.*, **47**, 192–205.

ALLEN RD AND ROBERTS TK. (1987). The role of spermine in the cytotoxic effects of seminal plasma. *Am. J. Reprod. Immunol. Microbiol.*, **13**, 4–8.

ALLOUB MI, BARR BBB, McLAREN KM, SMITH IW, BUNNEY MH AND SMART GE. (1989). Human papillomavirus infection and cervical intraepithelial neoplasia in women with renal allografts. *Br. Med. J.*, **298**, 153–156.

AVERILL-BATES DA, AGOSTINELLI E, PRZYBYTKOWSKI E, MATEESCU MA AND MONDOVI B. (1993). Cytotoxicity and kinetic analysis of purified bovine serum amine oxidase in the presence of spermine in chinese hamster ovary cells. *Arch. Biochem. Biophys.*, **300**, 75–79.

BANK CM, OFFERMANS JP, GIJZEN AH, SMITS F, VAN DIEIJEN-VISSER MP AND BROMBACHER PJ. (1991). Diamine oxidase activity in amniotic fluid for diagnosis of ruptured membranes. *Eur. J. Clin. Chem. Clin. Biochem.*, **29**, 743–748.

BOUZYK E AND ROSIEK O. (1988). Clastogenic and cytotoxic effects of spermine oxidation products in mouse lymphoma L5178Y cells. *Cancer Lett.*, **39**, 93–99.

BROE D, VAN DONGEN J, COWLEY D AND VACCA A. (1992). Detection of premature rupture of membranes by measuring diamine oxidase in vaginal fluid: false-negative results caused by obstetric antiseptic creams. *Clin. Chem.*, **38**, 784.

CHEN T-M AND DEFENDI V. (1992). Functional interaction of p53 with HPV18 E6, c-myc and H-ras in 3T3 cells. *Oncogene*, **7**, 1541–1547.

DE VILLIERS EM, SCHNEIDER A, MIKLAW H, PAPENDICK U, WAGNER D, WESH H, WAHRENDORF J AND ZUR HAUSEN H. (1987). Human papillomavirus infections in women with and without abnormal cervical cytology. *Lancet*, **2**, 703–706.

FERNANDEZ C, SHARRARD RM, MONKS N AND BARRETT CLR. (1994). Measurement of the oxidisable polyamine content of seminal plasma. *Clin. Chim. Acta*, **227**, 201–208.

FRANCO EL. (1991). Viral etiology of cervical cancer: a critique of the evidence. *Rev. Infect. Dis.*, **13**, 1195–1206.

GRAM IT, MACALUSO M AND STALSBERG H. (1992). Oral contraceptive use and the incidence of cervical intraepithelial neoplasia. *Am. J. Obstet. Gynecol.*, **167**, 40–44.

GRAMZINSKI RA, PARCHMENT RE AND PIERCE GB. (1990). Evidence linking programmed cell death in the blastocyst to polyamine oxidation. *Differentiation*, **43**, 59–65.

GUIJON FB, PARASKEVAS M AND BRUNHAM R. (1985). The association of sexually transmitted diseases with cervical intraepithelial neoplasia: a case-control study. *Am. J. Obstet. Gynecol.*, **151**, 185–190.

HENLE KJ, MOSS AJ AND NAGLE WA. (1986). Mechanisms of spermidine cytotoxicity at 37°C and 43°C in Chinese hamster ovary cells. *Cancer Res.*, **46**, 175–182.

HENRY MJ, STANLEY MW, CRUIKSHANK S AND CARSON L. (1989). Association of human immunodeficiency virus-induced immunosuppression with human papillomavirus infection and cervical intraepithelial neoplasia. *Am. J. Obstet. Gynecol.*, **160**, 352–353.

HERRERO R, BRINTON LA, REEVES WC, BRENES MM, TENORIO F, DE BRITTON RC, GAITAN E, GARCIA M AND RAWLS WE. (1990). Sexual behaviour, venereal diseases, hygiene practices, and invasive cervical cancer in a high-risk population. *Cancer*, **65**, 380–386.

HILDESHEIM A, MANN V, BRINTON LA, SZKLO M, REEVES WC AND RAWLS WE. (1991). Herpes simplex virus type 2: a possible interaction with human papillomavirus types 16/18 in the development of invasive cervical carcinoma. *Int. J. Cancer*, **49**, 335–340.

HILDESHEIM A, SCHIFFMAN MH, GRAVITT PE, GLASS AG, GREER CE, ZHANG T, SCOTT DR, RUSH BB, LAWLER P, SHERMAN ME, KURMAN RJ AND MANES MM. (1994). Persistence of type-specific human papillomavirus infection among cytologically normal women. *J. Infect. Dis.*, **169**, 235–240.

JAKOBSEN H, RUI H, THOMASSEN Y, HALD T AND PURVIS K. (1989). Polyamines and other accessory gland secretions in human seminal plasma 8 years after vasectomy. *J. Reprod. Fertil.*, **87**, 39–45.

JÄNNE J, HÖLTTÄ E, HAARANEN P AND ELFVING K. (1973). Polyamines and polyamine metabolising enzyme activities in human semen. *Clin. Chim. Acta*, **48**, 393–401.

JOFFE GP, FOXMAN B, SCHMIDT AJ, FARRIS KB, CARTER RJ, NEUMANN S, TOLO K-A AND WALTERS AM. (1992). Multiple partners and partner choice as risk factors for sexually transmitted disease among female college students. *Sex. Trans. Dis.*, **19**, 272–278.

JONES CJ, BRINTON LA, HAMMAN RF, STOLLEY PD, LEHMAN HF, LEVINE RS AND MALLIN K. (1990). Risk factors for *in situ* cervical cancer: results from a case-control study. *Cancer Res.*, **50**, 3657–3662.

KELLY RW, HOLLAND P, SKIBINSKI G, HARRISON C, MCMILLAN L, HARGREAVE TB AND JAMES K. (1991). Extracellular organelles (prostasomes) are immunosuppressive components of human semen. *Clin. Exp. Immunol.*, **86**, 550–556.

LA VECCHIA C, FRANCESCHI S, DECARLI A, FASOLI M, GENTILE A, PARAZZINI F AND REGALLO M. (1986). Sexual factors, venereal diseases, and the risk of intraepithelial and invasive cervical neoplasia. *Cancer*, **58**, 935–941.

MEANWELL CA, COX MF, BLACKLEDGE G AND MAITLAND NJ. (1987). HPV16 DNA in normal and malignant cervical epithelium: implications for the aetiology and behaviour of cervical neoplasia. *Lancet*, **1**, 703–707.

MITCHELL H, DRAKE M AND MEDLEY G. (1986). Prospective evaluation of risk of cervical cancer after cytological evidence on human papillomavirus infection. *Lancet*, **1**, 573–575.

OEFNER PJ, WONGYAI S AND BONN G. (1992). High-performance liquid chromatographic determination of free polyamines in human seminal plasma. *Clin. Chim. Acta*, **205**, 11–18.

PARCHMENT RE AND PIERCE GB. (1989). Polyamine oxidation, programmed cell death, and regulation of melanoma in the murine embryonic limb. *Cancer Res.*, **49**, 6680–6686.

POTISCHMAN N. (1993). Nutritional epidemiology of cervical neoplasia. *J. Nutr.*, **123**, 424–429.

QUAN CP, ROUX CR, PILLOT J AND BOUVET JP. (1990). Delineation between T and B suppressive molecules from human seminal plasma: II. Spermine is the major suppressor of T-lymphocytes *in vitro*. *Am. J. Reprod. Immunol.*, **22**, 64–69.

QUAN CP, D'AZAMBUJA S, PILLOT J AND BOUVET J-P. (1991). Protection by human serum from the immunosuppression induced by spermine in vitro. *Am. J. Reprod. Immunol.*, **25**, 153–157.

QUAYLE AJ, KELLY RW, HARGREAVE TB AND JAMES K. (1989). Immunosuppression by seminal prostaglandins. *Clin. Exp. Immunol.*, **75**, 387–391.

REEVES WC, CAUSSY D, BRINTON LA, BRENES MM, MONTALVAN P, GOMEZ B, DE BRITTON RC, MORICE E, GAITAN E, LOO DE LAO S AND RAWLS WE. (1987). Case-control study of human papillomaviruses and cervical cancer in Latin America. *Int. J. Cancer*, **40**, 450–454.

SCHEFFNER M, TAKAHASHI T, HUIBREGSTE JM, MINNA JD AND HOWLEY PM. (1992). Interaction of the human papillomavirus type 16 E6 oncoprotein with wild type and mutant human p53 proteins. *J. Virol.*, **66**, 5100–5105.

SCHIFFMAN, MH. (1992). Recent progress in defining the epidemiology of human papillomavirus infection and cervical neoplasia. *J. Natl Cancer Inst.*, **84**, 394–398.

SCHNEIDER A, HOTZ M AND GISSMAN L. (1987). Increased prevalence of human papillomaviruses in the lower genital tract of pregnant women. *Int. J. Cancer*, **40**, 198–201.

SHOHAT B, MAAYAN R, SINGER R, SAGIV M, KAUFMAN H AND ZUKERMAN Z. (1989). Immunosuppressive activity and polyamine levels of seminal plasma in azospermic, oligospermic, and normospermic men. *Arch. Androl.*, **24**, 41–50.

SINGER R, SAGIV M, LEVINSKY H, MAAYAN R, SEGENREICH E AND ALLALOUF D. (1989). The influence of seminal plasma and polyaminic substances on the motility of isolated human sperm. *Int. J. Fertil.*, **34**, 224–230.

SKIBINSKI G, KELLY RW, HARKISS D AND JAMES K. (1992). Immunosuppression by human seminal plasma – extracellular organelles (prostasomes) modulate activity of phagocytic cells. *Am. J. Reprod. Immunol.*, **28**, 97–103.

STIRDIVANT SM, HUBER HE, PATRICK DR, DEFEO-JONES D, MCAVOY E, GARSKY VM, OLIFF A AND HEIMBROOK DC. (1992). Human papillomavirus type 16 E7 protein inhibits DNA binding by the retinoblastoma gene product. *Mol. Cell Biol.*, **12**, 1905–1914.

VALLELY PJ, SHARRARD RM AND REES RC. (1988). The identification of factors in seminal plasma responsible for suppression of natural killer cell activity. *Immunology*, **63**, 451–456.

VAN DER LINDEN PJQ, KETS M, GIMPEL JA AND WIEGERINCK MAHM. (1992). Cyclic changes in the concentration of glucose and fructose in human cervical mucus. *Fertil. Steril.*, **57**, 573–577.

WILLIAMS-ASHMAN HG AND LOCKWOOD DH. (1970). Role of polyamines in reproductive physiology and sex hormone action. *Ann. NY. Acad. Sci.*, **171**, 882–894.

WINKELSTEIN W. (1990). Smoking and cervical cancer – current status: a review. *Am. J. Epidemiol.*, **131**, 945–957.

ZUR HAUSEN H. (1986). Intracellular surveillance of persisting viral infections. Human genital cancer results from deficient control of papillomavirus infection. *Lancet*, **2**, 489–491.


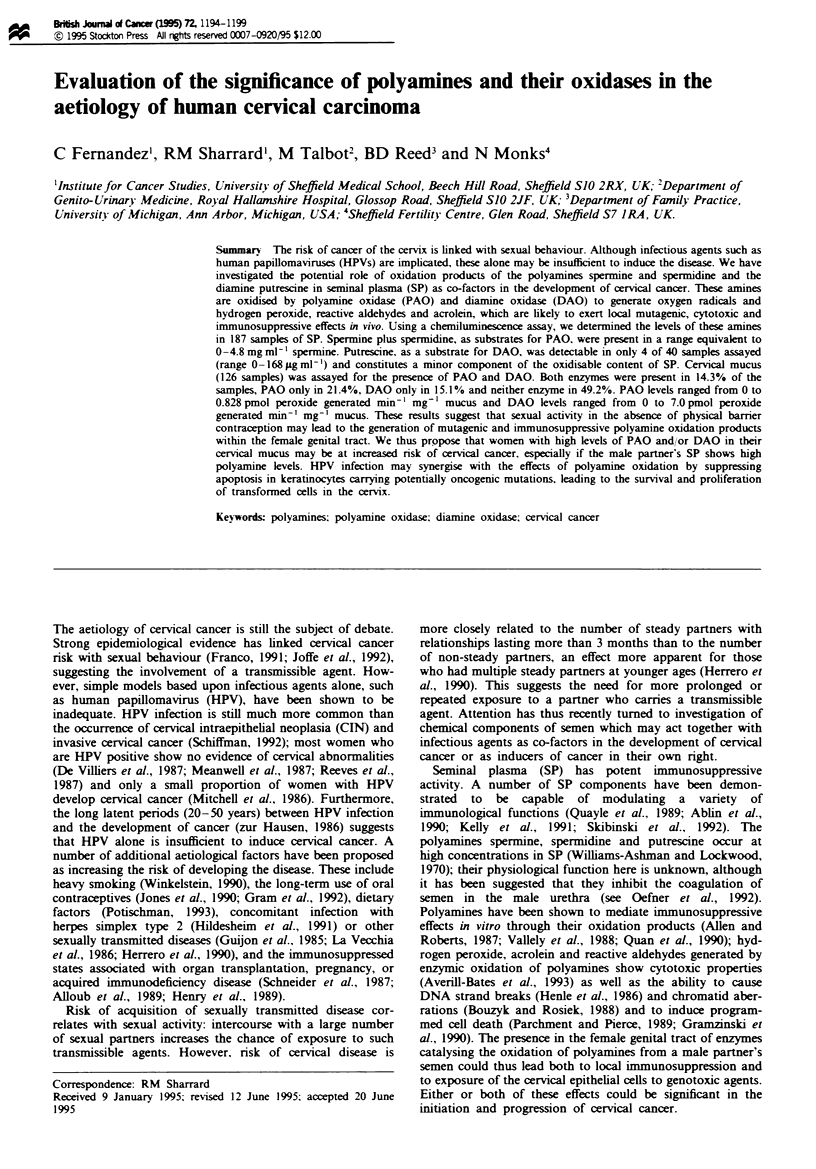

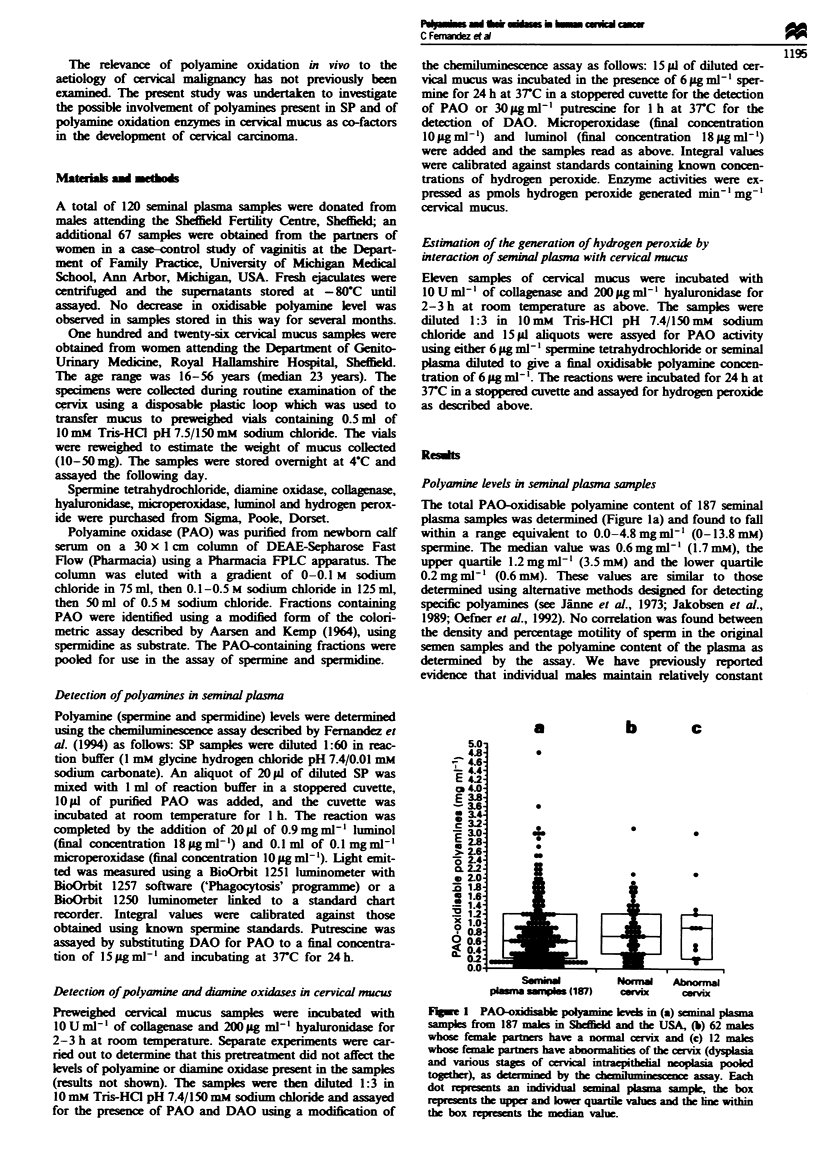

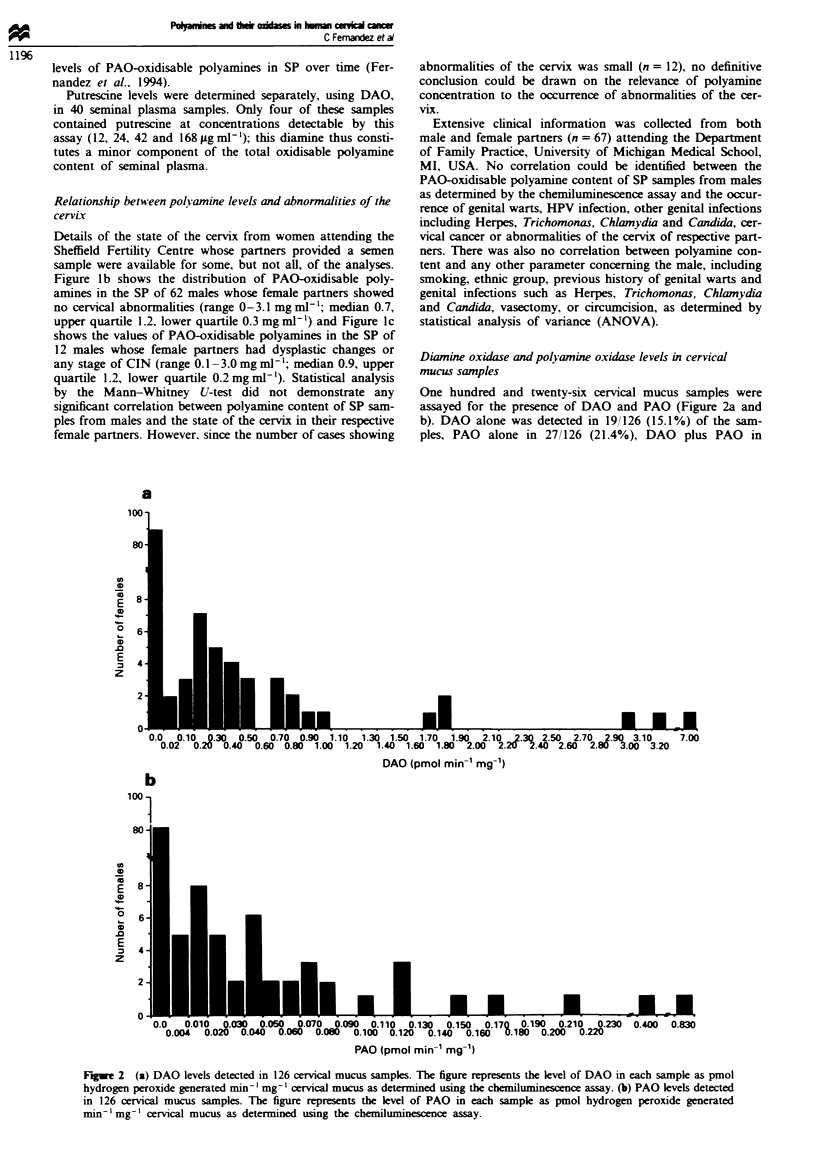

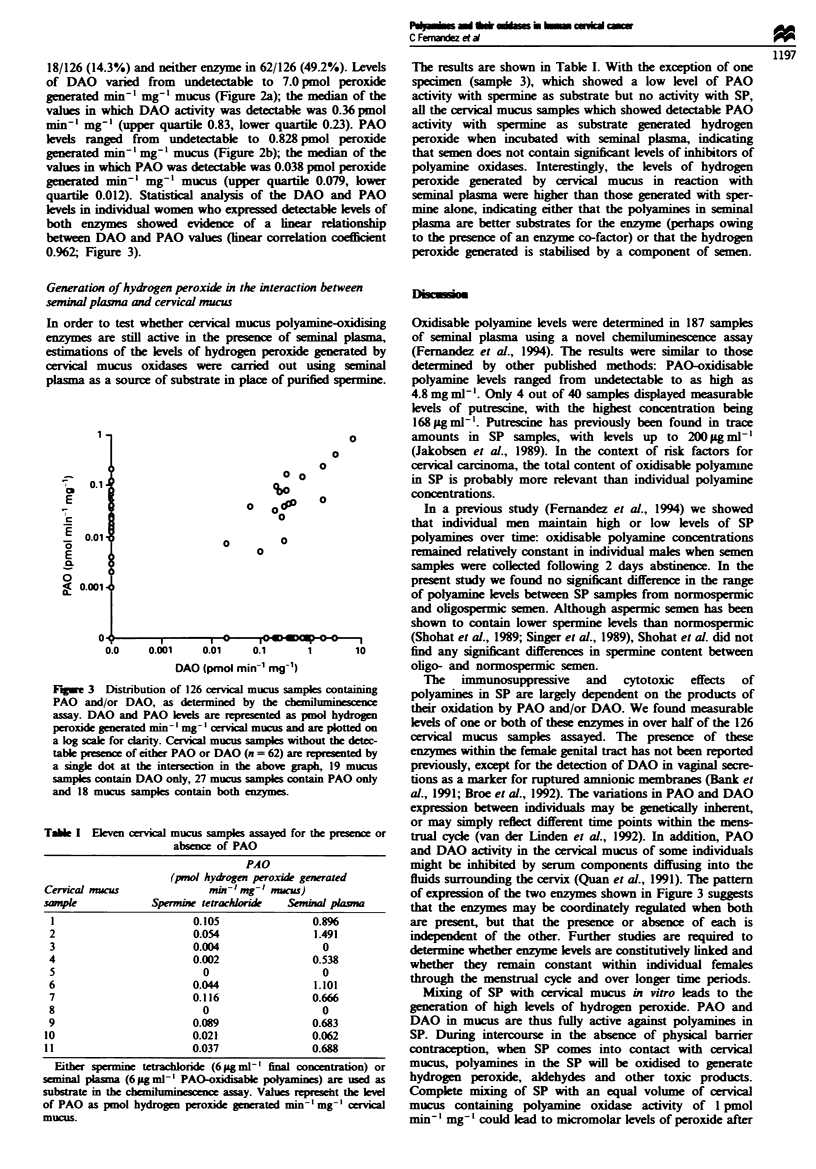

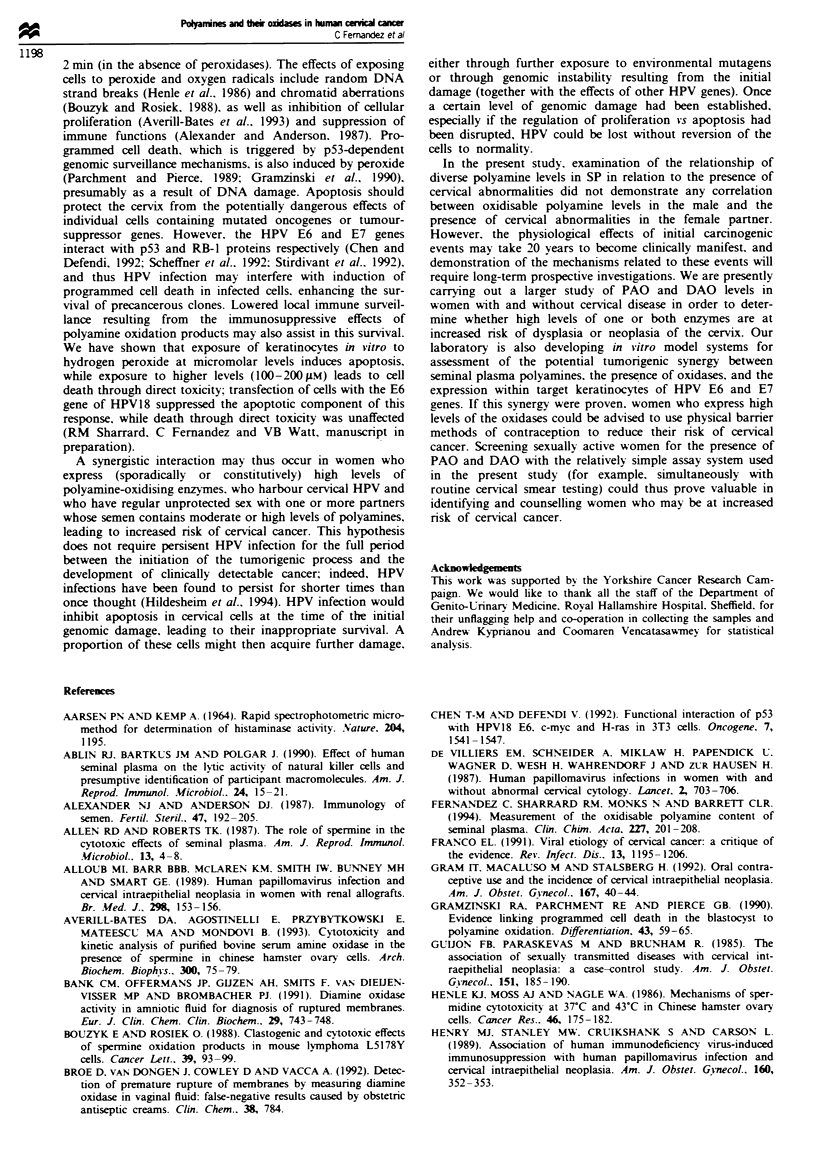

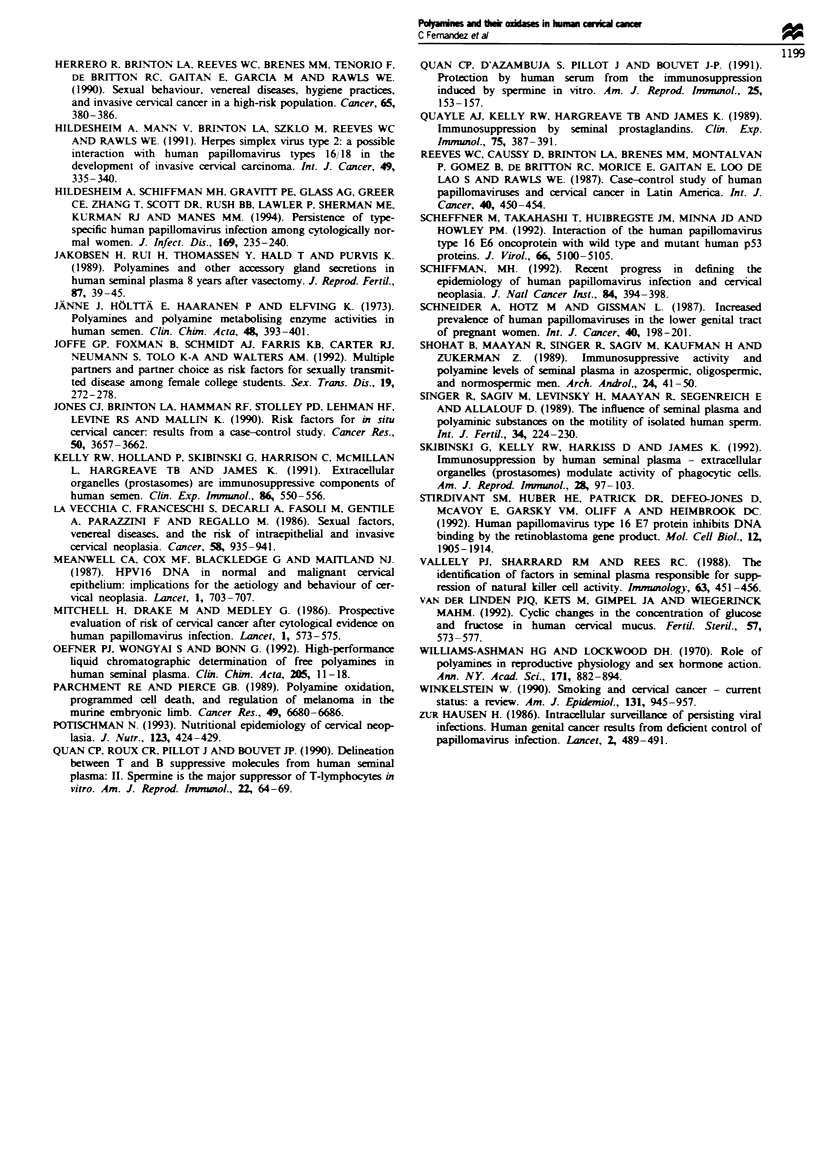


## References

[OCR_00718] AARSEN P. N. (1964). RAPID SPECTROPHOTOMETRIC MICROMETHOD FOR DETERMINATION OF HISTAMINASE ACTIVITY.. Nature.

[OCR_00723] Ablin R. J., Bartkus J. M., Polgár J. (1990). Effect of human seminal plasma on the lytic activity of natural killer cells and presumptive identification of participant macromolecules.. Am J Reprod Immunol.

[OCR_00729] Alexander N. J., Anderson D. J. (1987). Immunology of semen.. Fertil Steril.

[OCR_00739] Alloub M. I., Barr B. B., McLaren K. M., Smith I. W., Bunney M. H., Smart G. E. (1989). Human papillomavirus infection and cervical intraepithelial neoplasia in women with renal allografts.. BMJ.

[OCR_00744] Averill-Bates D. A., Agostinelli E., Przybytkowski E., Mateescu M. A., Mondovi B. (1993). Cytotoxicity and kinetic analysis of purified bovine serum amine oxidase in the presence of spermine in Chinese hamster ovary cells.. Arch Biochem Biophys.

[OCR_00751] Bank C. M., Offermans J. P., Gijzen A. H., Smits F., van Dieijen-Visser M. P., Brombacher P. J. (1991). Diamine oxidase activity in amniotic fluid for diagnosis of ruptured membranes.. Eur J Clin Chem Clin Biochem.

[OCR_00755] Bouzyk E., Rosiek O. (1988). Clastogenic and cytotoxic effects of spermine oxidation products in mouse lymphoma L5178Y cells.. Cancer Lett.

[OCR_00760] Broe D., Van Dongen J., Cowley D., Vacca A., Voreteliac V., Maquire D., Ellis V. (1992). Detection of premature rupture of membranes by measuring diamine oxidase in vaginal fluid: false-negative results caused by obstetric antiseptic creams.. Clin Chem.

[OCR_00766] Chen T. M., Defendi V. (1992). Functional interaction of p53 with HPV18 E6, c-myc and H-ras in 3T3 cells.. Oncogene.

[OCR_00777] Fernandez C., Sharrard R. M., Monks N., Barrett C. L. (1994). Measurement of the oxidisable polyamine content of seminal plasma.. Clin Chim Acta.

[OCR_00782] Franco E. L. (1991). Viral etiology of cervical cancer: a critique of the evidence.. Rev Infect Dis.

[OCR_00786] Gram I. T., Macaluso M., Stalsberg H. (1992). Oral contraceptive use and the incidence of cervical intraepithelial neoplasia.. Am J Obstet Gynecol.

[OCR_00791] Gramzinski R. A., Parchment R. E., Pierce G. B. (1990). Evidence linking programmed cell death in the blastocyst to polyamine oxidation.. Differentiation.

[OCR_00798] Guijon F. B., Paraskevas M., Brunham R. (1985). The association of sexually transmitted diseases with cervical intraepithelial neoplasia: a case-control study.. Am J Obstet Gynecol.

[OCR_00804] Henle K. J., Moss A. J., Nagle W. A. (1986). Mechanism of spermidine cytotoxicity at 37 degrees C and 43 degrees C in Chinese hamster ovary cells.. Cancer Res.

[OCR_00809] Henry M. J., Stanley M. W., Cruikshank S., Carson L. (1989). Association of human immunodeficiency virus-induced immunosuppression with human papillomavirus infection and cervical intraepithelial neoplasia.. Am J Obstet Gynecol.

[OCR_00819] Herrero R., Brinton L. A., Reeves W. C., Brenes M. M., Tenorio F., de Britton R. C., Gaitan E., Garcia M., Rawls W. E. (1990). Sexual behavior, venereal diseases, hygiene practices, and invasive cervical cancer in a high-risk population.. Cancer.

[OCR_00826] Hildesheim A., Mann V., Brinton L. A., Szklo M., Reeves W. C., Rawls W. E. (1991). Herpes simplex virus type 2: a possible interaction with human papillomavirus types 16/18 in the development of invasive cervical cancer.. Int J Cancer.

[OCR_00835] Hildesheim A., Schiffman M. H., Gravitt P. E., Glass A. G., Greer C. E., Zhang T., Scott D. R., Rush B. B., Lawler P., Sherman M. E. (1994). Persistence of type-specific human papillomavirus infection among cytologically normal women.. J Infect Dis.

[OCR_00842] Jakobsen H., Rui H., Thomassen Y., Hald T., Purvis K. (1989). Polyamines and other accessory sex gland secretions in human seminal plasma 8 years after vasectomy.. J Reprod Fertil.

[OCR_00854] Joffe G. P., Foxman B., Schmidt A. J., Farris K. B., Carter R. J., Neumann S., Tolo K. A., Walters A. M. (1992). Multiple partners and partner choice as risk factors for sexually transmitted disease among female college students.. Sex Transm Dis.

[OCR_00860] Jones C. J., Brinton L. A., Hamman R. F., Stolley P. D., Lehman H. F., Levine R. S., Mallin K. (1990). Risk factors for in situ cervical cancer: results from a case-control study.. Cancer Res.

[OCR_00848] Jänne J., Hölttä E., Haaranen P., Elfving K. (1973). Polyamines and polyamine-metabolizing enzyme activities in human semen.. Clin Chim Acta.

[OCR_00866] Kelly R. W., Holland P., Skibinski G., Harrison C., McMillan L., Hargreave T., James K. (1991). Extracellular organelles (prostasomes) are immunosuppressive components of human semen.. Clin Exp Immunol.

[OCR_00873] La Vecchia C., Franceschi S., Decarli A., Fasoli M., Gentile A., Parazzini F., Regallo M. (1986). Sexual factors, venereal diseases, and the risk of intraepithelial and invasive cervical neoplasia.. Cancer.

[OCR_00876] Meanwell C. A., Cox M. F., Blackledge G., Maitland N. J. (1987). HPV 16 DNA in normal and malignant cervical epithelium: implications for the aetiology and behaviour of cervical neoplasia.. Lancet.

[OCR_00884] Mitchell H., Drake M., Medley G. (1986). Prospective evaluation of risk of cervical cancer after cytological evidence of human papilloma virus infection.. Lancet.

[OCR_00887] Oefner P. J., Wongyai S., Bonn G. (1992). High-performance liquid chromatographic determination of free polyamines in human seminal plasma.. Clin Chim Acta.

[OCR_00894] Parchment R. E., Pierce G. B. (1989). Polyamine oxidation, programmed cell death, and regulation of melanoma in the murine embryonic limb.. Cancer Res.

[OCR_00897] Potischman N. (1993). Nutritional epidemiology of cervical neoplasia.. J Nutr.

[OCR_00909] Quan C. P., D'Azambuja S., Pillot J., Bouvet J. P. (1991). Protection by human serum from the immunosuppression induced by spermine in vitro.. Am J Reprod Immunol.

[OCR_00903] Quan C. P., Roux C., Pillot J., Bouvet J. P. (1990). Delineation between T and B suppressive molecules from human seminal plasma: II. Spermine is the major suppressor of T-lymphocytes in vitro.. Am J Reprod Immunol.

[OCR_00915] Quayle A. J., Kelly R. W., Hargreave T. B., James K. (1989). Immunosuppression by seminal prostaglandins.. Clin Exp Immunol.

[OCR_00920] Reeves W. C., Caussy D., Brinton L. A., Brenes M. M., Montalvan P., Gomez B., de Britton R. C., Morice E., Gaitan E., de Lao S. L. (1987). Case-control study of human papillomaviruses and cervical cancer in Latin America.. Int J Cancer.

[OCR_00925] Scheffner M., Takahashi T., Huibregtse J. M., Minna J. D., Howley P. M. (1992). Interaction of the human papillomavirus type 16 E6 oncoprotein with wild-type and mutant human p53 proteins.. J Virol.

[OCR_00931] Schiffman M. H. (1992). Recent progress in defining the epidemiology of human papillomavirus infection and cervical neoplasia.. J Natl Cancer Inst.

[OCR_00938] Schneider A., Hotz M., Gissmann L. (1987). Increased prevalence of human papillomaviruses in the lower genital tract of pregnant women.. Int J Cancer.

[OCR_00943] Shohat B., Maayan R., Singer R., Sagiv M., Kaufman H., Zukerman Z. (1990). Immunosuppressive activity and polyamine levels of seminal plasma in azo-ospermic, oligospermic, and normospermic men.. Arch Androl.

[OCR_00950] Singer R., Sagiv M., Levinsky H., Maayan R., Segenreich E., Allalouf D. (1989). The influence of seminal plasma and polyaminic substances on the motility of isolated human sperm.. Int J Fertil.

[OCR_00955] Skibinski G., Kelly R. W., Harkiss D., James K. (1992). Immunosuppression by human seminal plasma--extracellular organelles (prostasomes) modulate activity of phagocytic cells.. Am J Reprod Immunol.

[OCR_00962] Stirdivant S. M., Huber H. E., Patrick D. R., Defeo-Jones D., McAvoy E. M., Garsky V. M., Oliff A., Heimbrook D. C. (1992). Human papillomavirus type 16 E7 protein inhibits DNA binding by the retinoblastoma gene product.. Mol Cell Biol.

[OCR_00968] Vallely P. J., Sharrard R. M., Rees R. C. (1988). The identification of factors in seminal plasma responsible for suppression of natural killer cell activity.. Immunology.

[OCR_00981] Winkelstein W. (1990). Smoking and cervical cancer--current status: a review.. Am J Epidemiol.

[OCR_00774] de Villiers E. M., Wagner D., Schneider A., Wesch H., Miklaw H., Wahrendorf J., Papendick U., zur Hausen H. (1987). Human papillomavirus infections in women with and without abnormal cervical cytology.. Lancet.

[OCR_00972] van der Linden P. J., Kets M., Gimpel J. A., Wiegerinck M. A. (1992). Cyclic changes in the concentration of glucose and fructose in human cervical mucus.. Fertil Steril.

[OCR_00985] zur Hausen H. (1986). Intracellular surveillance of persisting viral infections. Human genital cancer results from deficient cellular control of papillomavirus gene expression.. Lancet.

